# Disproportionality analysis of oesophageal toxicity associated with oral bisphosphonates using the FAERS database (2004–2023)

**DOI:** 10.3389/fphar.2024.1473756

**Published:** 2024-11-07

**Authors:** Lin Chen, Zhicheng Dai, Huangrong Song, Jiafeng Zhang, Tuo Li

**Affiliations:** ^1^ Department of Cardiology, The First People’s Hospital of Xiaoshan District, Xiaoshan Affiliated Hospital of Wenzhou Medical University, Hangzhou, China; ^2^ Department of Orthopedics, Shanghai General Hospital, Shanghai Jiao Tong University School of Medicine, Shanghai, China; ^3^ Department of Reproductive Medicine, Shanghai Changzheng Hospital, Naval Medical University, Shanghai, China; ^4^ Department of Laboratory Medicine, Shanghai Changzheng Hospital, Naval Medical University, Shanghai, China; ^5^ Department of Endocrinology, Shanghai Changzheng Hospital, Naval Medical University, Shanghai, China

**Keywords:** adverse events, bisphosphonates, oesophageal, FAERS, disproportionality analysis

## Abstract

**Background:**

This study analyzed the FDA’s Adverse Event Reporting System (FAERS) data to investigate the correlation between oral bisphosphonates (BPs) and oesophageal adverse events (AEs).

**Methods:**

We systematically extracted data on adverse reactions to oral alendronate, risedronate, and ibandronate from the FAERS database, covering the period from the 2004 Q1 to the 2023 Q4. The role_code of AEs mainly includes primary suspect (PS), secondary suspect (SS), concomitant (C), and interaction (I). This study targeted reports with a role_code of “PS.” According to the FDA deduplication rule, the latest FDA_DT is selected when the CASEID is the same, and the higher PRIMARYID is selected when the CASEID and FDA_DT are the same. Our analysis leveraged four statistical methods, including the reporting odds ratio (ROR), proportional reporting ratio (PRR), Bayesian confidence propagation neural network (BCPNN), and the multi-item gamma Poisson shrinker (MGPS), to assess the relationship between oral bisphosphonates and oesophageal AEs. The Kaplan-Meier method was utilized to evaluate the cumulative incidence of oesophageal toxicity, while the log-rank test examined the temporal onset profiles of these toxicities. Additionally, the Pearson chi-squared test was employed to identify any significant differences in mortality and hospitalization rates associated with the oesophageal AEs caused by these medications.

**Results:**

The FAERS database had 41,590 AE reports for oral BPs, with 3,497 (8.41%) related to oesophageal AEs. Our findings indicate that oral BPs are disproportionately associated with an increased incidence of gastrointestinal system AEs at the system organ class (SOC) level. The adverse events identified at the preferred terms (PTs) level encompassed conditions such as gastroesophageal reflux disease, oesophagitis, and oesophageal pain. A significant divergence in the cumulative incidence of oesophageal AEs was observed among patients treated with the three different oral bisphosphonates, as confirmed by the log-rank test (*p* < 0.0001). Hospitalization rates varied significantly among patients receiving different BPs (*p* < 0.05), but no significant difference in mortality rates was found.

**Conclusion:**

The study establishes a significant link between oral BPs and oesophageal toxicity, highlighting the need for further research into the mechanisms of BP-induced oesophageal toxicity and potential preventive measures.

## Introduction

Bisphosphonates (BPs) are a class of compounds analogous to pyrophosphate, characterized by the presence of two phosphonate groups being linked to a central carbon atom (Lambrinoudaki et al., 2006). They are currently among the most extensively utilized pharmaceutical agents for the management of a spectrum of conditions, including Paget’s disease, osteoporosis, breast cancer, neoplastic bone metastases, multiple myeloma, select rare bone disorders, neurodegenerative diseases and dental applications (Peris et al., 2021; Shi et al., 2018; Zameer et al., 2018; Biggin and Munns, 2017; Ramaswamy and Shapiro, 2003; Coleman, 2020; Chapurlat and Legrand, 2021; Ralston, 2020; Sedghizadeh et al., 2021). The two main modes of administration of bisphosphonates are oral and intravenous (i.v.), of which alendronate, risedronate, and ibandronate, the three oral BPs mainly used for the treatment of osteoporosis, are commonly used in clinical practice (Papapetrou, 2009). A clinical trial of intravenous ibandronate (2 or 6 mg) in breast cancer patients revealed that serious adverse events potentially attributable to the study drug encompassed bone pain, pulmonary edema, and fatigue (Body et al., 2003). Moreover, numerous instances of renal toxicity have been documented with the newer generation of intravenous BPs, namely, pamidronate and zoledronic acid (Rosen et al., 2003; Chang et al., 2003).

Nonetheless, oral BPs are predominantly associated with a range of gastrointestinal adverse events (AEs), with esophagitis being a notable example (Zografos et al., 2009). The discourse surrounding oral BPs and their gastrointestinal ramifications remains contentious. Research by Psimma C et al. has demonstrated that the use of oral BPs can precipitate adverse effects on the oral mucosa (Psimma et al., 2022). Furthermore, a group of scholars has posited that oral BPs are associated with a spectrum of gastrointestinal conditions, including erosive esophagitis, gastritis, duodenitis, impaired healing, and hemorrhage stemming from oesophageal, gastric, or peptic ulcers (Parfitt and Driman, 2007; Peng et al., 2014; Modi et al., 2016). However, findings from extensive randomized controlled trials (RCTs) involving a multitude of participants have not corroborated an elevated incidence of upper gastrointestinal tract AEs in individuals administered bisphosphonates. Further research, studies involving patients who had ceased bisphosphonate treatment and were subsequently randomized to either blinded re-treatment with a bisphosphonate or a placebo revealed that the majority (exceeding 85%) were capable of resuming treatment without a discernible difference in AEs between the two groups (Cryer and Bauer, 2002). It is important to note that clinical trial data, often characterized by stringent inclusion criteria and a finite participant pool, may not accurately mirror the complexities of real-world clinical scenarios. To date, there exists a paucity of pharmacovigilance studies examining oesophageal toxicity after the introduction of oral bisphosphonates to the market, and the debate concerning the adverse effects of oral bisphosphonates. Additionally, comparative analyses of oesophageal toxicity among different oral BPs remain unexplored.

Given the above, it is imperative to investigate the correlation between diverse oral BPs and oesophageal AEs. The objective of this study was to leverage the standardized dataset from FAERS to evaluate the potential risk of oesophageal toxicity linked to three specific oral BPs: alendronate, risedronate, and ibandronate.

## Methods

### Study design and data source

The disproportionality analysis undertaken in this study was designed as a case/non-case study to quantify the relationship between oral BPs and oesophageal AEs. This approach entailed the determination of the ratio of target AEs associated with oral BPs (cases) relative to all other pharmaceuticals (non-cases) (Peng et al., 2020; Shu et al., 2023). A significant safety signal is identified when oral BPs demonstrate an increased likelihood of causing a target AE compared to other medications. The data for this analysis were derived from the FDA’s official platform, with AEs coded using PTs as per the Medical Dictionary for Regulatory Activities (MedDRA) (version 26.1). Furthermore, all relevant PTs, encompassing symptoms, signs, investigations, or diagnoses, were categorized into coherent groups using the Standardized MedDRA Queries (SMQs) to delineate the medical condition of interest. In this research, the focused SMQ for oesophageal toxicity encompassed 172 PTs (Supplementary Table 1). To incorporate the most recent case reports, data from the first quarter of 2004 to the fourth quarter of 2023, as recorded in the FAERS database, were retrieved and integrated into the study’s analysis.

### Data extraction

**FIGURE 1:**
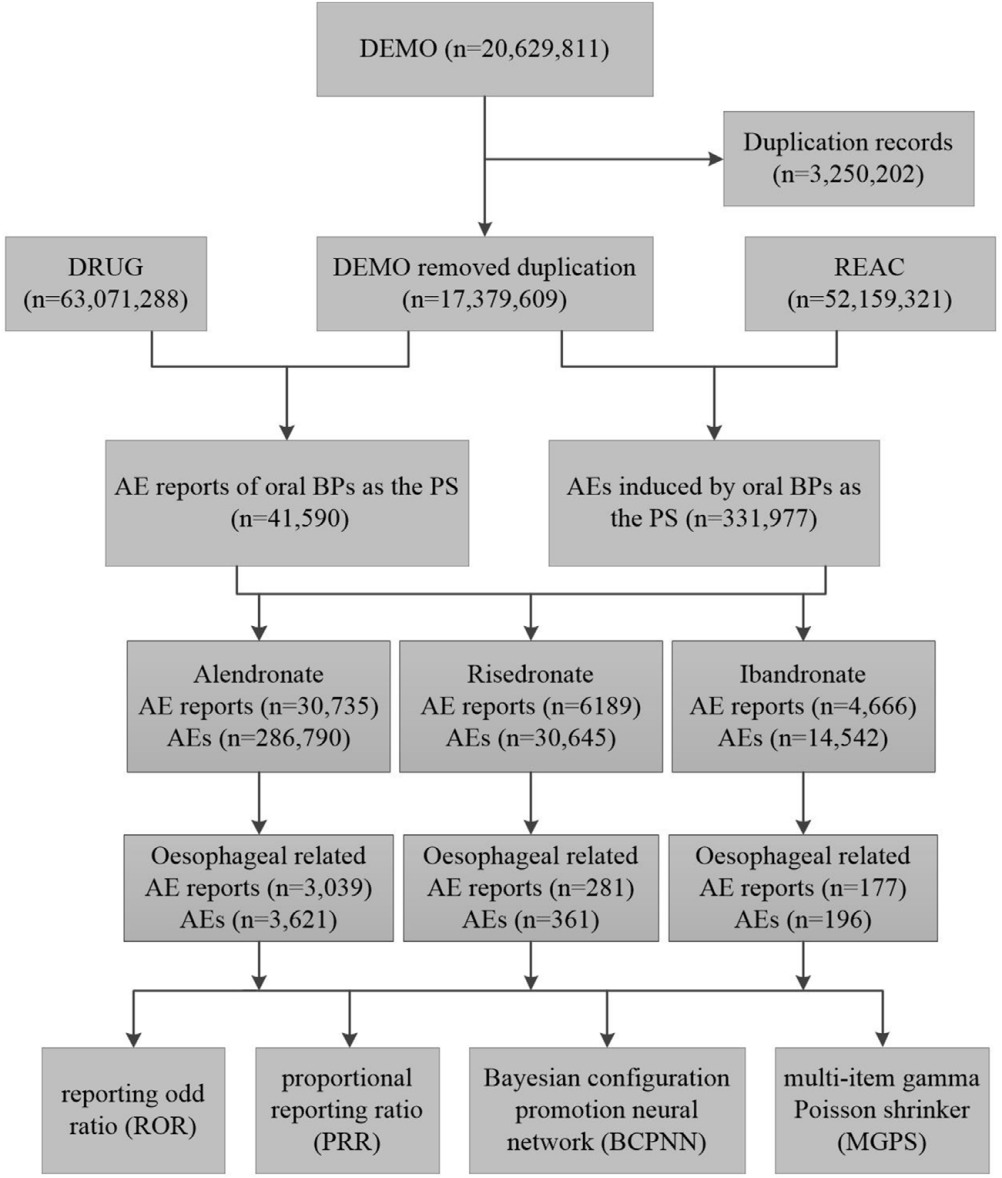
The flow diagram of selecting oral bisphosphonates-related oesophageal AEs from the FAERS database. Abbreviations: DEMO, demographic and administrative information; DRUG, drug information; REAC, preferred terminology for adverse event; PS, primary suspect drug; AEs, adverse events.

A deduplication protocol was meticulously implemented to safeguard the dataset’s uniqueness and integrity, adhering to the FDA’s stipulated guidelines. Cases pertinent to BPs were discerned by employing the search criteria “ALENDRONATE,” “RISEDRONATE,” and “IBANDRONATE,” with the designation “Primary suspected” (PS) applied to identify the role. Due to the prevalence of duplicative entries within FAERS, a deduplication protocol was implemented in accordance with FDA directives. According to the FDA’s recommended method for removing duplicate reports, the PRIMARYID, CASEID, and FDADT fields of the DEMO table are selected and sorted according to the order of CASEID, FDADT, and PRIMARYID, and the report with the same CASEID is retained with the largest FDA_DT value; followed by the report with the same CASEID and FDADT, and the report with the largest PRIMARYID value. For reports with the same CASEID, the one with the highest FDA_DT value is retained; secondly, for reports with the same CASEID and FDADT, the one with the highest PRIMARYID is retained (Huang, 2022; Giunchi et al., 2023). The procedural steps of the screening process are delineated in Figure 1.

### Disproportionality analysis

The application of a 2 × 2 contingency table for disproportionality analysis, as detailed in Supplementary Table 2, is a proven and efficacious method commonly utilized in the field of pharmacovigilance. This analytical framework was adopted in the current study to discern potential correlations between oral BPs and AEs. The detection of signals is predicated on the simultaneous application of four distinct algorithms: the ROR, the PRR, the BCPNN, and the MGPS, which collectively facilitate a thorough and robust assessment of observed associations (Hu et al., 2020; Huang et al., 2014). ROR and PRR methods are relatively simple to calculate and allow for an estimate of relative risk. Among these two methods, the PRR method can better control the impact of reporting biases and comorbid factors (Zhai et al., 2021). In order to avoid the high sensitivity of these two methods, this study combined signal detection methods such as PRR, ROR, BCPNN, and EBGM to screen overlapping signals, which can reduce the number of false positive and false negative signals with good sensitivity (Jaffa et al., 2015; Wang et al., 2021). For this study, AEs were deemed significant signals only when they satisfied the criteria set forth by all four algorithms simultaneously. The mathematical formulations and corresponding criteria for these algorithms are presented in Supplementary Table 3.

### Cumulative incidence and time-to-onset and statistical analysis

The time-to-onset was delineated as the interval commencing from the initiation of pharmaceutical therapy to the emergence of oesophageal toxicity. Consequently, the analysis was confined to reports that included time-to-onset data. The cumulative incidences of oesophageal toxicity were graphically represented using Kaplan-Meier method, with a log-rank test employed to discern differences in cumulative incidences between patients administered three different oral BPs. Continuous variables were expressed as mean ± standard deviation (SD), whereas categorical variables were presented as percentages. Chi-square test was applied to evaluate the disparity in mortality and hospitalization rates across various BPs. *p* < 0.05 was considered statistically significant. All data manipulation and statistical computations were performed using R software, version 4.2.2.

## Results

### Descriptive analysis

A total of 20,629,811 adverse drug reaction reports were recorded in the FAERS from the first quarter of 2004 to the fourth quarter of 2023. Among these, 41,590 pertained to oral BPs, with alendronate accounting for 30,735 reports, risedronate for 6,189, and ibandronate for 4,666. Reports of oesophageal -related AEs for alendronate, risedronate, and ibandronate were 3,039, 281, and 177, respectively (Figure 1). Alendronate was associated with the highest incidence of oesophageal AEs among the three oral BPs. The majority of these oesophageal toxicities were reported in females, with consumers and medical practitioners being the principal reporters, and the United States being the most frequently reported country of origin (Table 1). Analysis of the age distribution within the known population revealed a concentration of AEs within the 18–85 years age bracket (Table 1).

**TABLE 1:** The characteristics of oral bisphosphonates associated with oesophageal toxicity.

Characteristics	Alendronate N = 3,039	Risedronate N = 281	Ibandronate N = 177	Total N = 3,497
Sex				
Female	2,527 (83.2%)	243 (86.5%)	168 (94.9%)	2,938 (84.0%)
Male	210 (6.9%)	25 (8.9%)	4 (2.3%)	239 (6.8%)
Missing	302 (9.9%)	13 (4.6%)	5 (2.8%)	320 (9.2%)
Age (years)				
<18	2 (0.1%)	0 (0%)	0 (0%)	2 (0%)
18–64.9	635 (20.9%)	88 (31.3%)	59 (33.3%)	782 (22.4%)
65–85	618 (20.3%)	121 (43.1%)	53 (29.9%)	792 (22.6%)
>85	73 (2.4%)	12 (4.3%)	4 (2.3%)	89 (2.6%)
Missing	1711 (56.3%)	60 (21.4%)	61 (34.5%)	1832 (52.4%)
Mean ± SD	67.7 ± 12.7	68.0 ± 12.6	68.5 ± 11.7	67.9 ± 12.5
Reporters				
Consumer	1,112 (36.6%)	100 (35.6%)	119 (67.2%)	1,331 (38.1%)
Health Professional	42 (1.4%)	10 (3.6%)	2 (1.1%)	54 (1.5%)
Lawyer	72 (2.4%)	1 (0.4%)	6 (3.4%)	79 (2.3%)
Medical doctor	1,178 (38.8%)	70 (24.9%)	33 (18.6%)	1,281 (36.6%)
Other	435 (14.3%)	50 (17.8%)	8 (4.5%)	493 (14.1%)
Pharmacists	93 (3.1%)	21 (7.5%)	6 (3.4%)	120 (3.4%)
Missing	107 (3.5%)	29 (10.3%)	3 (1.7%)	139 (4.0%)
Reported Countries				
United States	2,531 (83.3%)	139 (49.5%)	156 (88.1%)	2,826 (80.8%)
United Kingdom	128 (4.2%)	13 (4.6%)	1 (0.6%)	142 (4.1%)
Canada	85 (2.8%)	46 (16.4%)	1 (0.6%)	132 (3.8%)
Australia	23 (0.8%)	12 (4.3%)	0 (0%)	35 (1.0%)
Japan	14 (0.5%)	20 (7.1%)	4 (2.3%)	38 (1.1%)
Other	258 (8.5%)	51 (18.1%)	15 (8.5%)	324 (9.3%)

### Analysis of the number of reports and time-to-onset analyses

**FIGURE 2:**
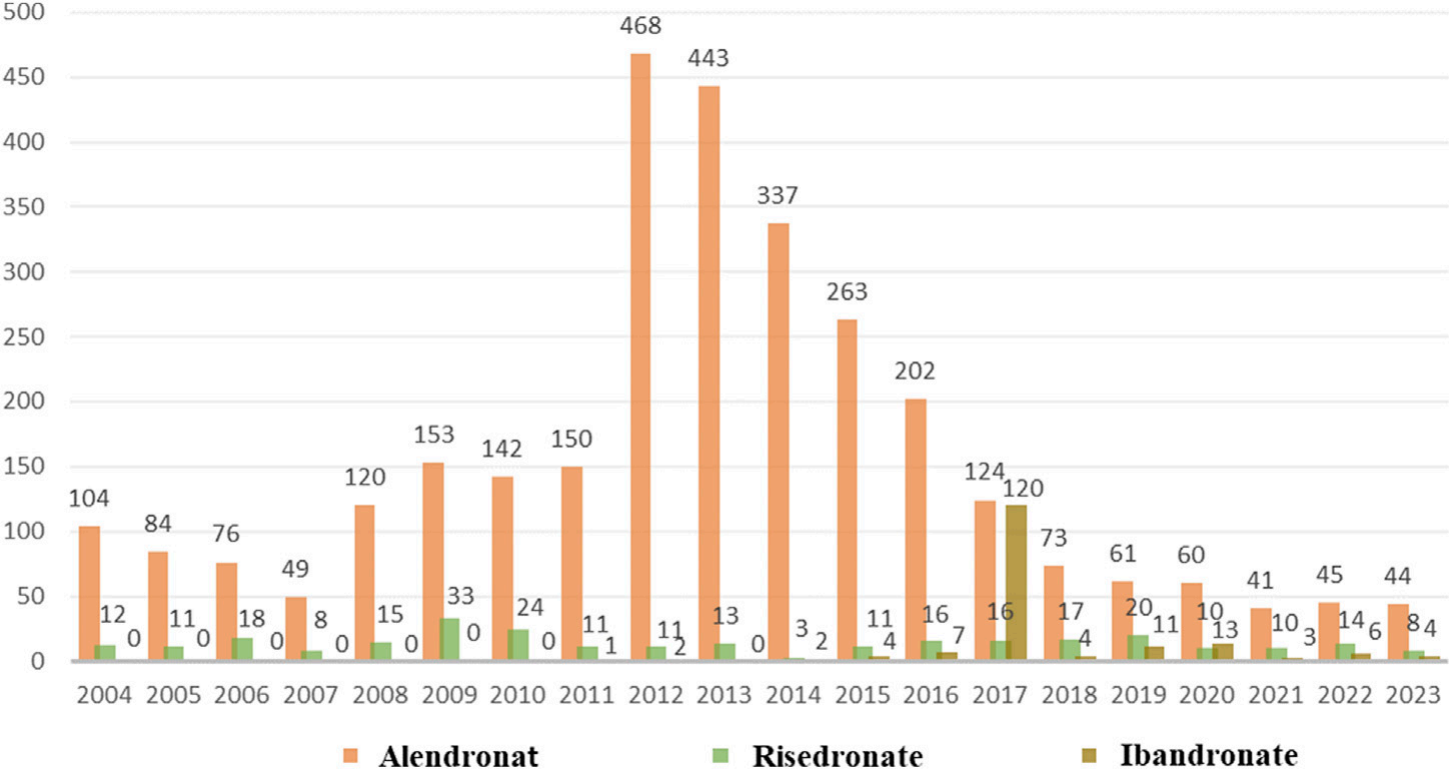
Number of AE reports of oesophageal toxicity caused by three oral bisphosphonates from the 2004 Q1 to the 2023 Q4.

**FIGURE 3:**
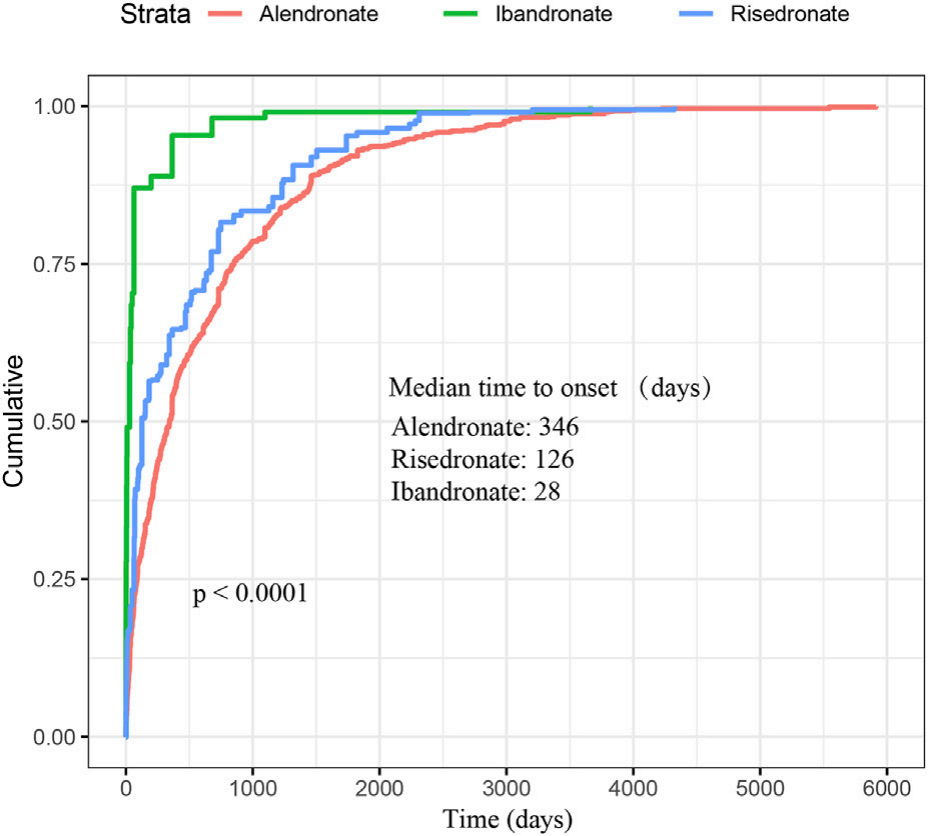
Cumulative distribution function of oral bisphosphonates by time-to-onset. This figure demonstrates the cumulative time to onset of oesophageal AEs associated with oral administration of the three bisphosphonates as well as the median time to onset. Significant difference was observed in the cumulative incidence of oesophageal AEs between patients treated with three oral bisphosphonates (log-rank test, *P* < 0.0001).

As depicted in Figure 2, a total of 3,497 cases of oesophageal toxicity induced by oral BPs were reported. The alendronate-related case reports were predominantly concentrated between 2008 and 2016, whereas the risedronate-related cases exhibited a generally stable trend. Ibandronate-related cases surged to 120 reports in 2017, with the remainder of the cases being more intermittent. Among the oesophageal toxicity AE reports associated with oral alendronate, risedronate, and ibandronate, time-to-onset data were available for 847, 88, and 19 reports, respectively. Kaplan-Meier curves illustrating the temporal profile of oesophageal toxicity are displayed in Figure 3. A marked difference was observed in the cumulative incidence of oesophageal AEs among patients treated with the three oral BPs, as evidenced by the log-rank test (*p* < 0.0001). The median times to onset for oesophageal toxicity associated with alendronate, risedronate, and ibandronate were 346, 126, and 28 days, respectively.

### Disproportionality analysis

**FIGURE 4:**
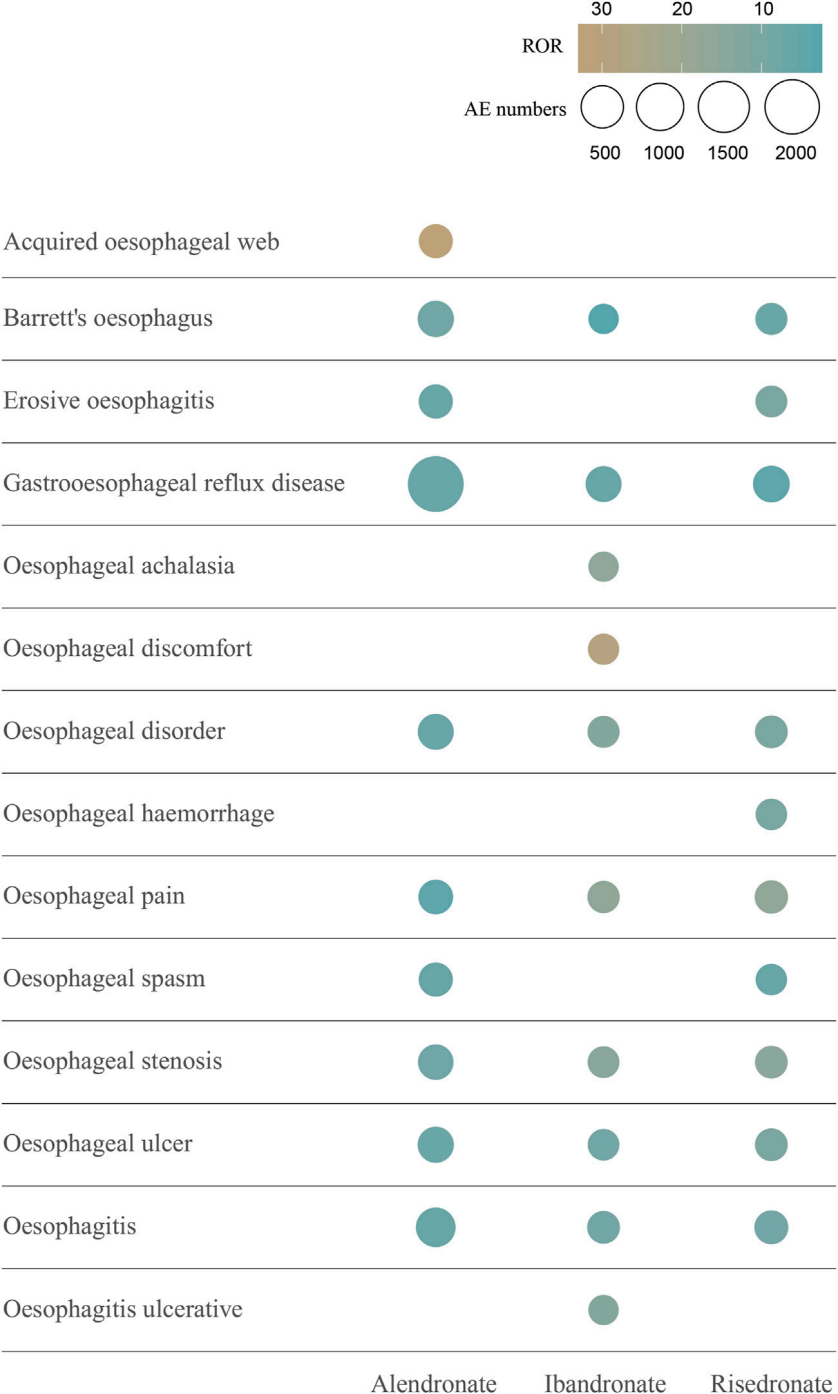
The top 10 AEs of oral bisphosphonates-related oesophageal toxicity ranked by AE numbers at the PT level. This figure shows the top 10 oesophageal toxicities in terms of number of occurrences associated with the 3 oral bisphosphonates. Different colours represent different ROR signal values. The size of the circles is proportional to the number of AEs. Abbreviations: AE, adverse event; ROR, reporting odds ratio; PT, preferred terms.

The signal values of AEs associated with oral alendronate, as classified by the SOC, are presented in Supplementary Table 4. Statistical analysis revealed that AEs linked to oral alendronate affected 27 organ systems, with 10 significant SOCs identified based on the fulfillment of at least one of the four computational criteria. A total of 30,645 AEs were attributed to oral risedronate, and 14,542 to oral ibandronate, both of which also impacted 27 organ systems (Supplementary Tables 5, 6). Notably, all three oral BPs exhibited positive signals in the category of Gastrointestinal disorders. The signal detection for oesophageal toxicity related to oral BPs is detailed in Table 2, with ibandronate demonstrating the most robust correlation with oesophageal toxicity (ROR = 7.03, PRR = 6.95, IC025 = 1.13, EBGM05 = 6.17). Additionally, the relationship between BPs and the PTs within the SMQs for oesophageal toxicity was examined (Figure 4). The top 10 PTs, ordered by the frequency of AE occurrences, were selected to investigate their correlation with each medication. Gastroesophageal reflux disease was identified as the PT most frequently associated with oesophageal toxicity across the three oral BPs. The PTs with the strongest associations for alendronate, risedronate, and ibandronate were acquired oesophageal web, oesophageal pain, and oesophageal discomfort, respectively, as determined by ROR values.

**TABLE 2:** Association of different oral bisphosphonates with oesophageal toxicity.

Drugs	AE numbers	ROR (95%Cl)	PRR (χ2)	IC (IC025)	EBGM (EBGM05)
Alendronate	3,621	*5.75 (5.56, 5.94)	*5.69 (13,123.20)	*2.47 (0.81)	*5.55 (5.39)
Risedronate	361	*5.74 (5.61, 6.38)	*5.69 (1,346.97)	*2.5 (0.84)	*5.67 (5.19)
Ibandronate	196	*7.03 (6.10, 8.11)	*6.95 (988.82)	*2.8 (1.13)	*6.94 (6.17)

This table shows the three bisphosphonates that are signal values for associated oesophageal toxicity. *Indicates statistically significant signals in algorithm. Abbreviations: AE, adverse event; ROR, reporting odds ratio; CI, confidence interval; PRR, proportional reporting ratio; χ2, chi-squared; IC, information component; IC025, the lower limit of the 95% CI, of the IC; EBGM05, empirical Bayesian geometric mean lower 95% CI, for the posterior distribution.

### Mortality and hospitalization rate due to oral BPs-related oesophageal toxicity

**FIGURE 5:**
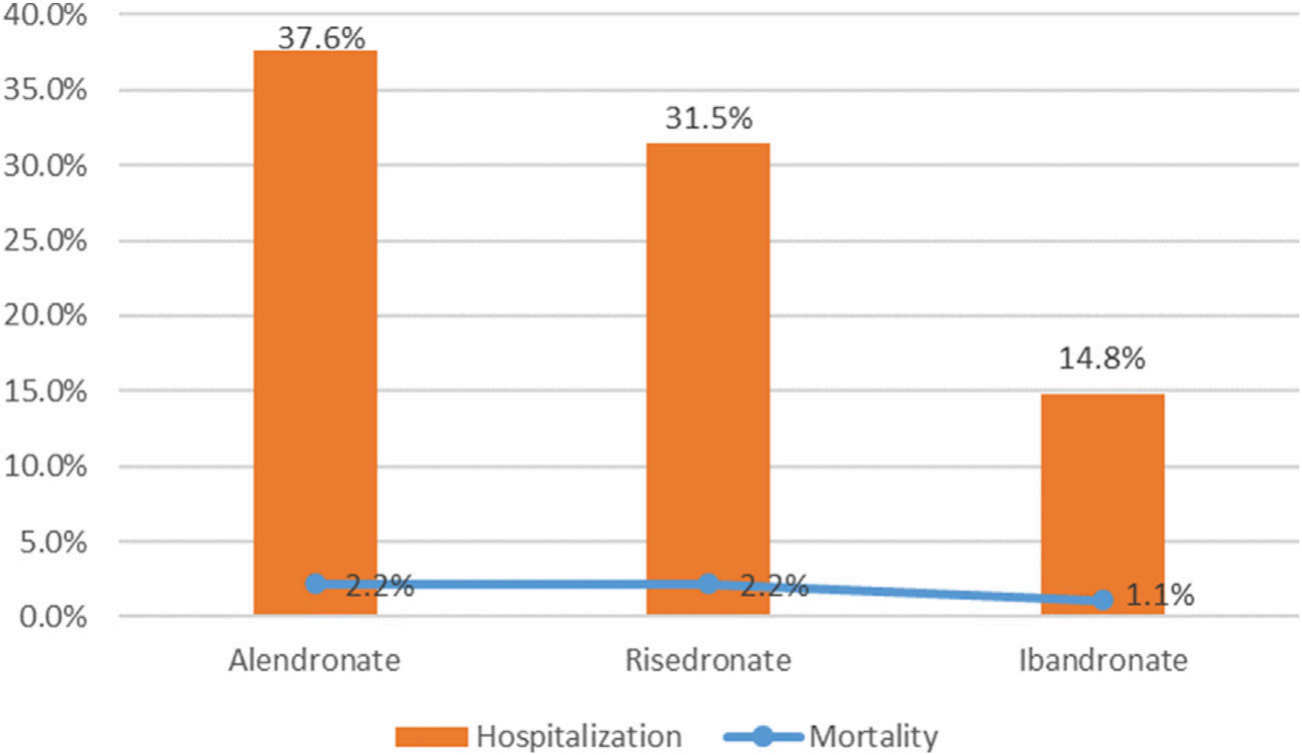
Hospitalization and mortality due to bisphosphonates-related oesophageal toxicity.

To evaluate the prognosis of oesophageal toxicity associated with oral BPs, an analysis was conducted on the proportion of patients who succumbed to death and those hospitalized (Figure 5). Among the hospitalization rates for oesophageal toxicity related to BPs, alendronate had the highest rate at 37.6%, followed by risedronate at 31.5% and ibandronate at 14.8%. The hospitalization rate for alendronate-induced oesophageal toxicity was significantly elevated compared to both risedronate and ibandronate (chi-squared test, *p* < 0.05). Additionally, a significant difference was observed between the hospitalization rates for risedronate and ibandronate (chi-squared test, *p* < 0.05). Mortality rates for patients on alendronate and risedronate were both 2.2%, while for ibandronate it was 1.1%. No significant disparity was detected among the drug-induced mortality rates (chi-squared test, *p* > 0.05).

## Discussion

Although oesophageal cancer linked to BPs has been extensively documented and scrutinized (Wright et al., 2015), a dearth of exhaustive research exists concerning the potential oesophageal toxicity related to oral BPs. To our knowledge, this study represents the most comprehensive analysis to date, leveraging the FAERS database from the 2004 Q1 to the 2023 Q4. The study’s objective was to explore the correlation between oesophageal toxicity and oral alendronate, risedronate, and ibandronate through disproportionality analysis and to assess variations among different BPs regarding their clinical profiles and outcomes in real-world settings.

Our research has revealed that three oral BPs are associated with oesophageal toxicity, with ibandronate exhibiting a more pronounced correlation (ROR = 7.03) than the other agents. Among the case reports of oesophageal toxicity, alendronate was implicated in 3,039 cases, while risedronate and ibandronate were associated with 281 and 177 cases, respectively. Descriptive studies indicate that oesophageal toxicity predominantly affects females, a trend potentially linked to the higher prevalence of BPs use among women, given the increased incidence of osteoporosis in this demographic, particularly postmenopausally (Bijelic et al., 2017). In the cohort experiencing risedronate-related oesophageal toxicity, the majority were over 65 years of age (47.4%), whereas, for the other two BPs, the majority of patients were within the 18–65 years age bracket. A comparative analysis of the cumulative incidence of oesophageal AEs among patients treated with the three oral BPs revealed a statistically significant divergence (*p* < 0.0001). Disproportionality analyses demonstrated an association between all three oral BPs and gastrointestinal disorders at the SOC level. Subsequent screenings based on SMQs identified an association between BPs and oesophageal toxicity, with oral alendronate specifically linked to oesophageal cancer, while the other two BPs showed no correlation with oesophageal malignancies. This association, however, is a subject of debate. A predominantly female cohort found no connection between oral bisphosphonates and upper gastrointestinal cancer (Morden et al., 2015). An epidemiological study within the UK’s General Practice Research Database, comparing the incidence of oesophageal and gastric cancer in patients exposed and not exposed to oral bisphosphonates, reported no elevated risk for either cancer (Cardwell et al., 2010). This contrasts with Green et al., who, using the same database, observed that one or more prescriptions for oral bisphosphonates raised the risk of oesophageal cancer by 30%, while 10 or more prescriptions nearly doubled this risk, with no increased risk for stomach or colorectal cancers (Green et al., 2010). Further validation is required through large, multicentre, prospective cohort studies to ascertain whether oral BPs can induce oesophageal cancer. The most common oesophageal toxicity associated with the three BPs was gastroesophageal reflux disease, succeeded by oesophagitis. A single-center, prospective cohort study involving 298 hospitalized adults actively taking risedronate or alendronate assessed upper gastrointestinal symptoms at baseline and 1–5 h post-administration. During follow-up, gastric and oesophageal symptoms affected 32 patients (10.7%), with epigastric burning, dysphagia, and regurgitation reported in 4.4% (n = 13), 3% (n = 9), and 2.7% (n = 8) patients, respectively, and heartburn, retrosternal pain, and odynophagia in 1.7% (n = 5), 1.7% (n = 5), and 0.3% (n = 1) patients (Nguyen et al., 2021). Concurrently, a study titled “Gastroesophageal reflux disease and oesophagitis associated with substance use: the modern state of the problem,” confirmed that bisphosphonates can cause gastroesophageal reflux disease and esophagitis (Osadchuk et al., 2019), reinforcing the reliability of our findings. In addition, among the top 10 PTs with the highest number of occurrences, Barrett’s oesophagus, gastroesophageal reflux disease, oesophageal disorder, oesophageal pain, oesophageal stenosis, oesophageal ulcer, and oesophagitis all appeared to be oesophageal adverse reactions occurring in the three oral BPs. Collectively, these findings underscore the necessity for clinicians to vigilantly monitor patients exhibiting oesophageal symptoms, particularly those concerning oesophageal malignancies when administering BPs.

To evaluate the prognosis of oesophageal toxicity related to oral antihypertensive medications, we examined the proportion of patients hospitalized and deceased as a consequence of oesophageal toxicity linked to the trio of oral BPs, ascertaining the presence of any statistical disparities. Statistical variance was observed among the hospitalization rates associated with each BP, with alendronate incurring the highest rate at 37.6%, succeeded by risedronate at 31.5%, and ibandronate at 14.8%. No significant difference was detected in the mortality rates attributable to the drugs. Consequently, when administering bisphosphonates, it is crucial to exercise caution to prevent hospitalization and death stemming from oesophageal toxicity induced by these oral medications.

The etiology of drug-induced oesophageal toxicity is complex, with three principal mechanisms identified from the current categorization of medications. These include agents that diminish the lower oesophageal sphincter pressure, such as anticholinergics (Koerselman et al., 1999; Ciccaglione et al., 2001) and benzodiazepines (e.g., diazepam) (Rushnak and Leevy, 1980), as well as aminophylline (theophylline) (Mungan and Pınarbaşı Şimşek, 2017); substances that exert a direct corrosive effect on the oesophageal mucosa and diminish its protective properties, including bisphosphonates (Grima et al., 2010; Ang et al., 2016) and a range of antibiotics (e.g., tetracycline, doxycycline, clindamycin, ciprofloxacin, ornidazole, rifampicin.) (Harirforoosh et al., 2013); and medications that delay gastric emptying, exemplified by calcium channel blockers (Findling et al., 1996). Given the direct corrosive potential of bisphosphonates on the oesophageal mucosa, it is recommended that these drugs be taken with a full glass of plain water (alendronate ≥200 mL, risedronate ≥120 mL, ibandronate ≥180 mL), and administered separately from other medications to minimize interaction. Patients on weekly bisphosphonate regimens should maintain an upright posture and abstain from eating for a minimum of 30 min; for ibandronate, this period should be extended to 60 min. This practice is intended to reduce the duration of contact between the medication and the oesophageal mucosa, thereby lessening the potential for damage (Vytrisalova et al., 2015; Wysowski, 2010).

Our observational study utilizing the FAERS database has inherent limitations common to spontaneous reporting systems. These include incomplete data, lack of comprehensive demographic and health information, reporting biases, and potential confounding factors like concurrent medication use. The voluntary nature of reporting also limits our ability to determine the total number of drug users, affecting the generalizability of our findings. Importantly, the disproportionality analysis results should not be interpreted as establishing causality between the drug and adverse events, as they do not account for confounding factors or alternative explanations. Nonetheless, our study provides valuable real-world insights into the oesophageal safety of oral bisphosphonates.

## Conclusion

Given the widespread utilization of bisphosphonates, safety concerns, notably concerning oesophageal adverse events, have emerged. In this study, leveraging the FAERS database, we conducted a thorough and systematic analysis of adverse reaction signals associated with three oral bisphosphonates. Our objective was to elucidate the link between bisphosphonate use and oesophageal toxicity, thereby offering insights to improve the clinical safety profile of these medications.

## Data Availability

Publicly available datasets were analyzed in this study. This data can be found here: https://www.fda.gov/drugs/drug-approvals-and-databases/fda-adverse-event-reporting-system-faers-database.

## Abbreviations

BPs, bisphosphonates; AEs, adverse events; FDA, Food and Drug Administration; FAERS, Food and Drug Administration Adverse Event Reporting System; ROR, reporting odds ratio; PRR, proportional reporting ratio; BCPNN, Bayesian configuration promotion neural network; MGPS, multi-item gamma Poisson shrinker; PS, primary suspect; PTs, Preferred Terms; SOC, system organ class.
