# Supplementary material for: Disproportionality analysis of oesophageal toxicity associated with oral bisphosphonates using the FAERS database (2004–2023)
Source: Front Pharmacol. 2024 Nov 7;15:1473756. doi: 10.3389/fphar.2024.1473756 (PMC11578700; doi:10.3389/fphar.2024.1473756)
Supplement: Supplementary file 1 [file Table1.DOCX]

**Table S1 The PT codes and names included in the SMQ of** **oesophageal toxicity.**

| PT codes | PT names | PT codes | PT names | PT codes | PT names |
| --- | --- | --- | --- | --- | --- |
| 10078506 | Lymphocytic oesophagitis | 10074242 | Herpes simplex oesophagitis | 10052488 | Oesophageal ulcer perforation |
| 10064212 | Eosinophilic oesophagitis | 10074243 | Varicella zoster oesophagitis | 10055668 | Necrotising oesophagitis |
| 10075253 | Atrio-oesophageal fistula | 10082996 | Oesophageal abscess | 10063655 | Erosive oesophagitis |
| 10030146 | Oesophageal atresia | 10030216 | Oesophagitis | 10080386 | Acute oesophageal mucosal lesion |
| 10079063 | Oesophageal cyst | 10030218 | Oesophagitis chemical | 10056091 | Varices oesophageal |
| 10086623 | Oculo-digito-oesophageal-duodenal syndrome | 10030219 | Oesophagitis haemorrhagic | 10004808 | Biopsy oesophagus |
| 10044310 | Tracheo-oesophageal fistula | 10048899 | Radiation oesophagitis | 10004809 | Biopsy oesophagus abnormal |
| 10021612 | Incarcerated hiatus hernia | 10085026 | Immune-mediated oesophagitis | 10004810 | Biopsy oesophagus normal |
| 10067357 | Hiatus hernia, obstructive | 10017885 | Gastrooesophageal reflux disease | 10030222 | Oesophagoscopy |
| 10013563 | Diverticulum oesophageal | 10067752 | Oesophageal hypomotility | 10030223 | Oesophagoscopy abnormal |
| 10072785 | Pharyngo-oesophageal diverticulum | 10071554 | Oesophageal atony | 10030224 | Oesophagoscopy normal |
| 10013544 | Diverticulitis oesophageal | 10088653 | Gastrooesophageal reflux in neonate | 10053057 | Oesophagogastroduodenoscopy |
| 10004428 | Benign oesophageal neoplasm | 10013924 | Dyskinesia oesophageal | 10053058 | Oesophagogastroscopy |
| 10051935 | Oesophageal polyp | 10030136 | Oesophageal achalasia | 10053059 | Oesophagram |
| 10071192 | Oesophageal papilloma | 10060696 | Presbyoesophagus | 10053061 | Oesophageal motility test |
| 10000582 | Acquired tracheo-oesophageal fistula | 10072419 | Oesophageal motility disorder | 10072163 | Oesophagogastroduodenoscopy abnormal |
| 10056992 | Oesophagobronchial fistula | 10030184 | Oesophageal spasm | 10072164 | Oesophagogastroduodenoscopy normal |
| 10065835 | Oesophageal fistula | 10089269 | Oesophageal hypermotility | 10072165 | Oesophagogastroscopy normal |
| 10066870 | Aorto-oesophageal fistula | 10061882 | Oesophageal neoplasm | 10072166 | Oesophagogastroscopy abnormal |
| 10083015 | Oesophageal-pulmonary fistula | 10030137 | Oesophageal adenocarcinoma | 10088340 | Computerised tomogram oesophagus abnormal |
| 10084038 | Oesophagomediastinal fistula | 10030140 | Oesophageal adenocarcinoma recurrent | 10050703 | Oesophageal manometry |
| 10006707 | Burn oesophageal | 10030141 | Oesophageal adenocarcinoma stage 0 | 10063566 | Oesophageal pH |
| 10024389 | Leukoplakia oesophageal | 10030142 | Oesophageal adenocarcinoma stage I | 10064172 | Oesophageal pH increased |
| 10030164 | Oesophageal dilatation | 10030143 | Oesophageal adenocarcinoma stage II | 10064173 | Oesophageal pH decreased |
| 10057003 | Oesophageal mass | 10030144 | Oesophageal adenocarcinoma stage III | 10066597 | Gastrooesophageal variceal haemorrhage prophylaxis |
| 10058522 | Oesophageal injury | 10030145 | Oesophageal adenocarcinoma stage IV | 10064064 | Gastrooesophageal reflux prophylaxis |
| 10061318 | Oesophageal disorder | 10030159 | Oesophageal carcinoma recurrent | 10081862 | Lower oesophageal sphincter magnetic augmentation |
| 10064342 | Oesophageal oedema | 10030187 | Oesophageal squamous cell carcinoma recurrent | 10076443 | Radiotherapy to oesophagus |
| 10072280 | Oesophageal mucosa erythema | 10030188 | Oesophageal squamous cell carcinoma stage 0 | 10057024 | Oesophagogastrectomy |
| 10072350 | Oesophageal mucosal dissection | 10030189 | Oesophageal squamous cell carcinoma stage I | 10030174 | Oesophageal lesion excision |
| 10076952 | Feline oesophagus | 10030190 | Oesophageal squamous cell carcinoma stage II | 10030207 | Oesophageal variceal injection |
| 10077019 | Oesophageal mucosal blister | 10030192 | Oesophageal squamous cell carcinoma stage IV | 10030208 | Oesophageal variceal ligation |
| 10077822 | Oesophageal mucosal tear | 10055102 | Oesophageal cancer metastatic | 10030215 | Oesophagectomy |
| 10087414 | Oesophageal hyperkeratosis | 10058527 | Oesophageal squamous cell carcinoma metastatic | 10030221 | Oesophagoenterostomy |
| 10088436 | Oesophageal wall hypertrophy | 10061534 | Oesophageal squamous cell carcinoma | 10038333 | Removal of foreign body from oesophagus |
| 10004137 | Barrett's oesophagus | 10082968 | Oesophageal adenosquamous carcinoma | 10050898 | Oesophageal tamponade |
| 10066141 | Oesophageal mucosal hyperplasia | 10030178 | Oesophageal obstruction | 10053056 | Oesophagostomy |
| 10030172 | Oesophageal haemorrhage | 10030194 | Oesophageal stenosis | 10053060 | Oesophageal graft |
| 10030210 | Oesophageal varices haemorrhage | 10052820 | Acquired oesophageal web | 10057305 | Oesophageal dilation procedure |
| 10077486 | Oesophageal intramural haematoma | 10072208 | Oesophageal fibrosis | 10058381 | Oesophageal fistula repair |
| 10088347 | Gastrooesophageal haemorrhage | 10074074 | Oesophageal compression | 10058453 | Oesophageal anastomosis |
| 10030154 | Oesophageal candidiasis | 10081525 | Oesophageal stent stenosis | 10061319 | Oesophageal operation |
| 10030200 | Oesophageal tuberculosis | 10030180 | Oesophageal pain | 10061985 | Oesophagoplasty |
| 10049018 | Cytomegalovirus oesophagitis | 10053634 | Oesophageal discomfort | 10068839 | Oesophagogastrostomy |
| 10049656 | Fungal oesophagitis | 10065567 | Oesophageal food impaction | 10069955 | Oesophageal stent removal |
| 10052330 | Herpes oesophagitis | 10030181 | Oesophageal perforation | 10072018 | Radiofrequency ablation of oesophagus |
| 10056704 | Parasitic oesophagitis | 10030201 | Oesophageal ulcer | 10078775 | Oesophageal prosthesis insertion |
| 10058803 | Oesophageal infection | 10030202 | Oesophageal ulcer haemorrhage | 10078812 | Oesophageal polypectomy |
| 10060190 | Viral oesophagitis | 10049098 | Oesophagitis ulcerative | 10082992 | Oesophageal bypass |
| 10061320 | Oesophagitis bacterial | 10052211 | Oesophageal rupture | 10089638 | Hiatal hernia gangrenous |
| 10076368 | Feeding tube user | 10070818 | Oesophageal irritation | 10030191 | Oesophageal squamous cell carcinoma stage III |
| 10010564 | Congenital oesophageal stenosis | 10062879 | Gastrooesophageal sphincter insufficiency | 10048852 | Oesophagogastric fundoplasty |
| 10010565 | Congenital oesophageal web | 10062878 | Gastrooesophageal cancer | 10007861 | Celestin tube insertion |
| 10021530 | Imperforate oesophagus | 10081398 | Gastrooesophageal cancer recurrent | 10007862 | Celestin tube removal |
| 10061069 | Congenital oesophageal anomaly | 10051671 | Metastases to oesophagus | 10051918 | Oesophagocardiomyotomy |
| 10086585 | Neuroendocrine carcinoma of the oesophagus | 10030155 | Oesophageal carcinoma | 10072786 | Tracheo-oesophageal puncture |
| 10077873 | Oesophagopleural fistula | 10030162 | Oesophageal carcinoma stage 0 | 10068197 | Gastric tube reconstruction |
| 10050171 | Oesophageal dysplasia |  |  |  |  |

This table shows 172 relevant PT codes and names for oesophageal toxicity according to the SMQ. Abbreviations: PT, preferred term; SMQ, Standardized MedDRA Queries;
