# Supplementary material for: Disproportionality analysis of oesophageal toxicity associated with oral bisphosphonates using the FAERS database (2004–2023)
Source: Front Pharmacol. 2024 Nov 7;15:1473756. doi: 10.3389/fphar.2024.1473756 (PMC11578700; doi:10.3389/fphar.2024.1473756)
Supplement: Supplementary file 2 [file Table2.DOCX]

**Table S2 Two-by-two contingency table for disproportionality analyses.**

|  | Target AEs | Non-Target AEs | Total |
| --- | --- | --- | --- |
| Target drug | a | b | a+b |
| All other drugs | c | d | c+d |
| Total | a+c | b+d | N=a+b+c+d |

Abbreviations: AEs; adverse events
