# Supplementary material for: Disproportionality analysis of oesophageal toxicity associated with oral bisphosphonates using the FAERS database (2004–2023)
Source: Front Pharmacol. 2024 Nov 7;15:1473756. doi: 10.3389/fphar.2024.1473756 (PMC11578700; doi:10.3389/fphar.2024.1473756)
Supplement: Supplementary file 3 [file Table5.DOCX]

**Table S5 The signal strength of reports associated with risedronate at the SOC level in the FAERS database.**

| SOC Name | AE numbers | ROR (95%Cl) | PRR (***χ*^2^**) | IC (IC025) | EBGM (EBGM05) |
| --- | --- | --- | --- | --- | --- |
| Product issues | 135 | 0.28 (0.24 - 0.33) | 0.28 (251.04) | -1.82 (-3.49) | 0.28 (0.23) |
| Metabolism and nutrition disorders | 447 | 0.67 (0.61 - 0.73) | 0.67 (73.7) | -0.57 (-2.24) | 0.67 (0.61) |
| Ear and labyrinth disorders | 165 | *1.24 (1.06 - 1.44) | 1.23 (7.39) | 0.30 (-1.36) | 1.23 (1.06) |
| Hepatobiliary disorders | 273 | 0.98 (0.87 - 1.1) | 0.98 (0.15) | -0.03 (-1.70) | 0.98 (0.86) |
| Infections and infestations | 1255 | 0.77 (0.73 - 0.82) | 0.78 (79.44) | -0.35 (-2.02) | 0.78 (0.74) |
| Investigations | 1761 | 0.92 (0.88 - 0.97) | 0.93 (10.85) | -0.10 (-1.78) | 0.93 (0.88) |
| Nervous system disorders | 1720 | 0.64 (0.61 - 0.67) | 0.66 (337.14) | -0.60 (-2.27) | 0.66 (0.62) |
| Injury, poisoning, and procedural complications | 3486 | *1.14 (1.1 - 1.18) | 1.13 (53.91) | 0.17 (-1.50) | 1.13 (1.09) |
| Musculoskeletal and connective tissue disorders | 6589 | *4.95 (4.82 - 5.09) | *4.1 (16287.08) | *2.03 (0.37) | *4.1 (3.99) |
| Surgical and medical procedures | 299 | 0.74 (0.66 - 0.83) | 0.74 (26.77) | -0.42 (-2.09) | 0.74 (0.66) |
| Congenital, familial and genetic disorders | 46 | 0.47 (0.35 - 0.63) | 0.47 (26.87) | -1.07 (-2.74) | 0.47 (0.35) |
| Respiratory, thoracic, and mediastinal disorders | 961 | 0.66 (0.61 - 0.7) | 0.67 (168.97) | -0.58 (-2.25) | 0.67 (0.62) |
| Psychiatric disorders | 682 | 0.38 (0.35 - 0.41) | 0.39 (689.78) | -1.35 (-3.02) | 0.39 (0.36) |
| Neoplasms benign, malignant, and unspecified (Incl Cysts and Polyps) | 365 | 0.44 (0.39 - 0.49) | 0.44 (260.7) | -1.17 (-2.84) | 0.44 (0.4) |
| Immune system disorders | 267 | 0.79 (0.7 - 0.89) | 0.79 (14.69) | -0.33 (-2.00) | 0.79 (0.7) |
| Endocrine disorders | 59 | 0.76 (0.59 - 0.98) | 0.76 (4.39) | -0.39 (-2.06) | 0.76 (0.59) |
| Skin and subcutaneous tissue disorders | 804 | 0.48 (0.44 - 0.51) | 0.49 (454.12) | -1.03 (-2.70) | 0.49 (0.45) |
| General disorders and administration site conditions | 4456 | 0.81 (0.78 - 0.83) | 0.83 (177.74) | -0.26 (-1.93) | 0.83 (0.8) |
| Pregnancy, puerperium, and perinatal conditions | 27 | 0.2 (0.14 - 0.29) | 0.2 (85.21) | -2.30 (-3.97) | 0.2 (0.13) |
| Social circumstances | 327 | *2.32 (2.08 - 2.59) | *2.31 (242.45) | 1.20 (-0.46) | 2.3 (2.07) |
| Renal and urinary disorders | 328 | 0.55 (0.49 - 0.61) | 0.55 (119.58) | -0.85 (-2.52) | 0.55 (0.5) |
| Reproductive system and breast disorders | 94 | 0.34 (0.28 - 0.41) | 0.34 (121.12) | -1.55 (-3.22) | 0.34 (0.27) |
| Gastrointestinal disorders | 4196 | *1.7 (1.65 - 1.76) | 1.61 (1052.17) | 0.68 (-0.98) | 1.61 (0.56) |
| Cardiac disorders | 493 | 0.6 (0.55 - 0.65) | 0.61 (130.45) | -0.72 (-2.39) | 0.61 (0.55) |
| Vascular disorders | 373 | 0.56 (0.51 - 0.62) | 0.57 (127.43) | -0.82 (-2.49) | 0.57 (0.51) |
| Blood and lymphatic system disorders | 294 | 0.57 (0.5 - 0.63) | 0.57 (97.48) | -0.81 (-2.48) | 0.57 (0.5) |
| Eye disorders | 743 | *1.24 (1.15 - 1.33) | 1.23 (32.55) | 0.30 (-1.37) | 1.23 (1.14) |

*Indicates statistically significant signals in algorithm. Abbreviations: SOC, system organ class; AE, adverse event; ROR, reporting odds ratio; CI, confidence interval; PRR, proportional reporting ratio; χ2, chi-squared; IC, information component; IC025, the lower limit of the 95% CI of the IC; EBGM05, empirical Bayesian geometric mean lower 95% CI for the posterior distribution.
