# Supplementary material for: Disproportionality analysis of oesophageal toxicity associated with oral bisphosphonates using the FAERS database (2004–2023)
Source: Front Pharmacol. 2024 Nov 7;15:1473756. doi: 10.3389/fphar.2024.1473756 (PMC11578700; doi:10.3389/fphar.2024.1473756)
Supplement: Supplementary file 4 [file Table3.DOCX]

**Table S3 Four different types of disproportionality analysis.**

| **Algorithms** | | **Equation** | **Criteria** |
| --- | --- | --- | --- |
| ROR | ROR = (a/c)/(b/d)  95%CI=eln^(ROR)±1.96(1/a+1/b+1/c+1/d)^0.5^ | | 95% CI (lower limit) > 1, a ≥ 3 |
| PRR | | PRR=[a/(a+b)]/[c/(c+d)]  χ^2^ =[(ad-bc)^2^ (a+b + c + d)] / [(a+b) (c + d) (a+c) (b + d)] | PRR ≥ 2, χ^2^ ≥ 4, a ≥ 3 |
| BCPNN  MGPS | | IC=log_2_a(a+b+c+d)/((a+c) (a+b))  IC025=e^ln(IC)−1.96(1/a+1/b+1/c+1/d)^0.5^  EBGM=a(a+b+c+d)/((a+c) (a+b))  EBGM05=e^ln(EBGM)−1.64(1/a+1/b+1/c+1/d)^0.5^ | IC025 > 0, a ≥ 3  EBGM05 > 2, a＞0 |

Notes: Equation: a, number of reports containing both the target drug and the target adverse drug reaction; b, number of reports containing other adverse drug reactions of the target drug; c, number of reports containing the target adverse drug reaction of other drugs; d, number of reports containing other drugs and other adverse drug reactions. The MGPS employs an empirical Bayesian approach, whereby maximum likelihood estimates obtain a prior distribution, and the prior and likelihood are combined to obtain a posterior distribution. The fifth percentile of the posterior distribution is denoted by “EBGM05” and is interpreted as the one-sided 95% confidence lower bound for the EBGM. Abbreviations: ROR, reporting odds ratio; CI, confidence interval; χ2, chi-squared. PRR, proportional reporting ratio; BCPNN, Bayesian confidence propagation neural network; IC, information component; IC025, the lower limit of the 95% CI of the IC; MGPS, multiple gamma Poisson shrinkage; EBGM, empirical Bayesian geometric mean; EBGM05, empirical Bayesian geometric mean lower 95% CI for the posterior distribution.
