# Supplementary material for: Disproportionality analysis of oesophageal toxicity associated with oral bisphosphonates using the FAERS database (2004–2023)
Source: Front Pharmacol. 2024 Nov 7;15:1473756. doi: 10.3389/fphar.2024.1473756 (PMC11578700; doi:10.3389/fphar.2024.1473756)
Supplement: Supplementary file 5 [file Table4.DOCX]

**Table S4 The signal strength of reports associated with alendronate at the SOC level in the FAERS database.**

| SOC Name | AE numbers | ROR (95%Cl) | PRR (***χ*^2^**) | IC (IC025) | EBGM (EBGM05) |
| --- | --- | --- | --- | --- | --- |
| Surgical and medical procedures | 15707 | *4.44 (4.37 - 4.51) | *4.25 (38637.36) | *2.06 (0.4) | *4.17 (4.11) |
| Injury, poisoning, and procedural complications | 42082 | *1.53 (1.52 - 1.55) | 1.45 (6603.71) | 0.54 (-1.13) | 1.45 (1.44) |
| Gastrointestinal disorders | 28986 | *1.21 (1.19 - 1.22) | 1.19 (931.15) | 0.25 (-1.42) | 1.19 (1.17) |
| Musculoskeletal and connective tissue disorders | 55659 | *4.42 (4.38 - 4.46) | *3.75 (116255.55) | *1.89 (0.22) | *3.7 (3.66) |
| Respiratory, thoracic, and mediastinal disorders | 10188 | 0.74 (0.73 - 0.76) | 0.75 (858.73) | -0.41 (-2.07) | 0.75 (0.74) |
| Neoplasms benign, malignant, and unspecified (Incl Cysts and Polyps) | 4462 | 0.57 (0.56 - 0.59) | 0.58 (1397.77) | -0.78 (-2.45) | 0.58 (0.56) |
| Investigations | 11124 | 0.61 (0.60 - 062) | 0.62 (2671.92) | -0.68 (-2.34) | 0.63 (0.61) |
| Psychiatric disorders | 9465 | 0.56 (0.55 - 0.57) | 0.58 (3097.43) | -0.79 (-2.46) | 0.58 (0.57) |
| Product issues | 1329 | 0.29 (0.28 - 0.31) | 0.30 (2265.65) | -1.75 (-3.42) | 0.3 (0.28) |
| General disorders and administration site conditions | 24740 | 0.45 (0.44 - 0.45) | 0.49 (15526.16) | -1.01 (-2.68) | 0.49 (0.49) |
| Metabolism and nutrition disorders | 8725 | *1.42 (1.39 - 1.45) | 1.40 (1023.61) | 0.49 (-1.18) | 1.4 (1.37) |
| Vascular disorders | 7189 | *1.17 (1.14 - 1.20) | 1.17 (171.46) | 0.22 (-1.45) | 1.16 (1.14) |
| Skin and subcutaneous tissue disorders | 7508 | 0.47 (0.46 - 0.48) | 0.49 (4288.44) | -1.04 (-2.7) | 0.49 (0.48) |
| Infections and infestations | 15512 | *1.04 (1.02 - 1.05) | 1.03 (18.98) | 0.05 (-1.62) | 1.03 (1.02) |
| Renal and urinary disorders | 4718 | 0.85 (0.83 - 0.87) | 0.85 (123.10) | -0.23 (-1.9) | 0.85 (0.83) |
| Reproductive system and breast disorders | 2944 | *1.14 (1.10 - 1.18) | 1.14 (51.38) | 0.19 (-1.48) | 1.14 (1.1) |
| Nervous system disorders | 12855 | 0.50 (0.49 - 0.51) | 0.52 (6087.50) | -0.93 (-2.6) | 0.52 (0.52) |
| Blood and lymphatic system disorders | 3896 | 0.80 (0.78 - 0.83) | 0.81 (185.68) | -0.31 (-1.98) | 0.81 (0.78) |
| Immune system disorders | 2140 | 0.68 (0.65 - 0.70) | 0.68 (331.08) | -0.56 (-2.23) | 0.68 (0.65) |
| Cardiac disorders | 6850 | 0.90 (0.87 - 0.92) | 0.90 (80.87) | -0.15 (-1.82) | 0.9 (0.88) |
| Eye disorders | 3865 | 0.68 (0.66 - 0.70) | 0.68 (580.78) | -0.55 (-2.21) | 0.68 (0.66) |
| Endocrine disorders | 1975 | *2.77 (2.64 - 2.89) | *2.75 (2177.20) | 1.45 (-0.22) | *2.73 (2.61) |
| Ear and labyrinth disorders | 1862 | *1.50 (1.43 - 1.57) | 1.49 (301.78) | 0.57 (-1.09) | 1.49 (1.42) |
| Social circumstances | 834 | 0.63 (0.58 - 0.67) | 0.63 (185.97) | -0.67 (-2.34) | 0.63 (0.59) |
| Hepatobiliary disorders | 1538 | 0.58 (0.56 - 0.61) | 0.59 (450.54) | -0.77 (-2.43) | 0.59 (0.56) |
| Congenital, familial and genetic disorders | 507 | 0.56 (0.51 - 0.61) | 0.56 (178.12) | -0.84 (-2.51) | 0.56 (0.51) |
| Pregnancy, puerperium, and perinatal conditions | 130 | 0.10 (0.09 - 0.12) | 0.10 (1011.13) | -3.26 (-4.93) | 0.1 (0.09) |

*Indicates statistically significant signals in algorithm. Abbreviations: SOC, system organ class; AE, adverse event; ROR, reporting odds ratio; CI, confidence interval; PRR, proportional reporting ratio; χ2, chi-squared; IC, information component; IC025, the lower limit of the 95% CI of the IC; EBGM05, empirical Bayesian geometric mean lower 95% CI for the posterior distribution.
