# Supplementary material for: Disproportionality analysis of oesophageal toxicity associated with oral bisphosphonates using the FAERS database (2004–2023)
Source: Front Pharmacol. 2024 Nov 7;15:1473756. doi: 10.3389/fphar.2024.1473756 (PMC11578700; doi:10.3389/fphar.2024.1473756)
Supplement: Supplementary file 6 [file Table6.DOCX]

**Table S6 The signal strength of reports associated with ibandronate at the SOC level in the FAERS database.**

| SOC Name | AE numbers | ROR (95%Cl) | PRR (***χ*^2^**) | IC (IC025) | EBGM (EBGM05) |
| --- | --- | --- | --- | --- | --- |
| Blood and lymphatic system disorders | 81 | 0.33 (0.26 - 0.41) | 0.33 (111.73) | -1.60 (-3.26) | 0.33 (0.28) |
| Cardiac disorders | 178 | 0.45 (0.39 - 0.53) | 0.46 (115.58) | -1.12 (-2.78) | 0.46 (0.41) |
| Congenital, familial and genetic disorders | 20 | 0.43 (0.28 - 0.67) | 0.43 (14.75) | -1.20 (-2.87) | 0.43 (0.3) |
| Ear and labyrinth disorders | 90 | *1.42 (1.16 - 1.75) | 1.42 (11.2) | 0.51 (-1.16) | 1.42 (1.19) |
| Endocrine disorders | 19 | 0.52 (0.33 - 0.81) | 0.52 (8.58) | -0.95 (-2.62) | 0.52 (0.36) |
| Eye disorders | 202 | 0.7 (0.61 - 0.8) | 0.7 (25.54) | -0.51 (-2.17) | 0.7 (0.63) |
| Gastrointestinal disorders | 2457 | *2.18 (2.09 - 2.28) | 1.98 (1309.04) | 0.99 (-0.68) | 1.98 (1.91) |
| General disorders and administration site conditions | 2066 | 0.78 (0.75 - 0.82) | 0.82 (104.92) | -0.29 (-1.96) | 0.82 (0.78) |
| Hepatobiliary disorders | 42 | 0.31 (0.23 - 0.43) | 0.32 (62.52) | -1.66 (-3.32) | 0.32 (0.25) |
| Immune system disorders | 52 | 0.32 (0.25 - 0.42) | 0.33 (73.63) | -1.62 (-3.29) | 0.33 (0.26) |
| Infections and infestations | 542 | 0.7 (0.64 - 0.76) | 0.71 (66.15) | -0.49 (-2.15) | 0.71 (0.66) |
| Injury, poisoning, and procedural complications | 1567 | *1.07 (1.02 - 1.13) | 1.07 (7.06) | 0.09 (-1.57) | 1.07 (1.02) |
| Investigations | 466 | 0.5 (0.46 - 0.55) | 0.52 (224.45) | -0.95 (-2.62) | 0.52 (0.48) |
| Metabolism and nutrition disorders | 218 | 0.68 (0.6 - 0.78) | 0.69 (31.12) | -0.54 (-2.2) | 0.69 (0.62) |
| Musculoskeletal and connective tissue disorders | 3409 | *5.53 (5.33 - 5.75) | 4.47 (9682.41) | *2.16 (0.49) | *4.47 (4.33) |
| Neoplasms benign, malignant, and unspecified (Incl Cysts and Polyps) | 173 | 0.44 (0.38 - 0.51) | 0.44 (123.91) | -1.17 (-2.84) | 0.44 (0.39) |
| Nervous system disorders | 853 | 0.67 (0.62 - 0.71) | 0.69 (133.5) | -0.54 (-2.21) | 0.69 (0.65) |
| Pregnancy, puerperium, and perinatal conditions | 2 | 0.03 (0.01 - 0.13) | 0.03 (59.6) | -4.98 (-6.65) | 0.03 (0.01) |
| Product issues | 56 | 0.24 (0.19 - 0.32) | 0.25 (131.14) | -2.02 (-3.69) | 0.25 (0.2) |
| Psychiatric disorders | 658 | 0.78 (0.72 - 0.85) | 0.79 (37.6) | -0.33 (-2.00) | 0.79 (0.74) |
| Renal and urinary disorders | 199 | 0.71 (0.61 - 0.81) | 0.71 (24.18) | -0.50 (-2.16) | 0.71 (0.63) |
| Reproductive system and breast disorders | 41 | 0.31 (0.23 - 0.42) | 0.31 (62.36) | -1.68 (-3.34) | 0.31 (0.24) |
| Respiratory, thoracic, and mediastinal disorders | 421 | 0.6 (0.55 - 0.66) | 0.61 (106.59) | -0.70 (-2.37) | 0.61 (0.57) |
| Skin and subcutaneous tissue disorders | 394 | 0.49 (0.44 - 0.54) | 0.5 (202.16) | -0.99 (-2.65) | 0.5 (0.46) |
| Social circumstances | 46 | 0.68 (0.51 - 0.91) | 0.68 (6.81) | -0.55 (-2.22) | 0.68 (0.54) |
| Surgical and medical procedures | 137 | 0.72 (0.6 - 0.85) | 0.72 (15.36) | -0.48 (-2.14) | 0.72 (0.62) |
| Vascular disorders | 153 | 0.48 (0.41 - 0.57) | 0.49 (83.66) | -1.03 (-2.7) | 0.49 (0.43) |

*Indicates statistically significant signals in algorithm. Abbreviations: SOC, system organ class; AE, adverse event; ROR, reporting odds ratio; CI, confidence interval; PRR, proportional reporting ratio; χ2, chi-squared; IC, information component; IC025, the lower limit of the 95% CI of the IC; EBGM05, empirical Bayesian geometric mean lower 95% CI for the posterior distribution.
